# Comparative Analysis of Reproductive Traits in Black-Chinned Tilapia Females from Various Coastal Marine, Estuarine and Freshwater Ecosystems

**DOI:** 10.1371/journal.pone.0029464

**Published:** 2012-01-13

**Authors:** Moussa Guèye, Mbaye Tine, Justin Kantoussan, Papa Ndiaye, Omar Thiom Thiaw, Jean-Jacques Albaret

**Affiliations:** 1 IRD, route des hydrocarbures, Dakar, Sénégal; 2 Max Planck Institute for Molecular Genetics, Berlin, Germany; 3 IRD, LEMAR - Institut Universitaire Européen de la Mer, Plouzané, France; 4 Institut Fondamental d'Afrique Noire, Université Cheikh Anta Diop, Dakar, Senegal; 5 Laboratoire de Biologie cellulaire et moléculaire, Reproduction et Génétique, Université Cheikh Anta Diop de Dakar, Faculté des Sciences et Techniques, Dakar-Fann, Sénégal; Ecole Normale Supérieure de Lyon, France

## Abstract

The black-chinned tilapia *Sarotherodon melanotheron* is a marine teleost characterised by an extreme euryhalinity. However, beyond a certain threshold at very high salinity, the species exhibits impaired growth and precocious reproduction. In this study, the relationships between reproductive parameters, environmental salinity and condition factor were investigated in wild populations of this species that were sampled in two consecutive years (2003 and 2004) from three locations in Senegal with different salinities: Guiers lake (freshwater, 0 psu), Hann bay (seawater, 37 psu) and Saloum estuary (hypersaline water, 66–127 psu). The highest absolute fecundity and spawning weight were recorded in seawater by comparison to either freshwater or hypersaline water whereas the poorest condition factors were observed in the most saline sampling site. These results reflect higher resource allocation to the reproduction due to the lowest costs of adaptation to salinity in seawater (the natural environment of this species) rather than differences in food resources at sites and/or efficiency at foraging and prey availability. Fecundities, oocyte size as well as spawning weight were consistent from year to year. However, the relative fecundity in the Saloum estuary varied significantly between the dry and rainy raisons with higher values in the wet season, which seems to reflect seasonal variations in environmental salinity. Such a reproductive tactic of producing large amounts of eggs in the rainy season when the salinity in the estuary was lower, would give the fry a better chance at survival and therefore assures a high larval recruitment. An inverse correlation was found between relative fecundity and oocyte size at the two extreme salinity locations, indicating that *S. melanotheron* has different reproductive strategies in these ecosystems. The adaptive significance of these two reproductive modes is discussed in regard to the heavy osmotic constraint imposed by extreme salinities and high inter-specific competition.

## Introduction

**Figure 1:**
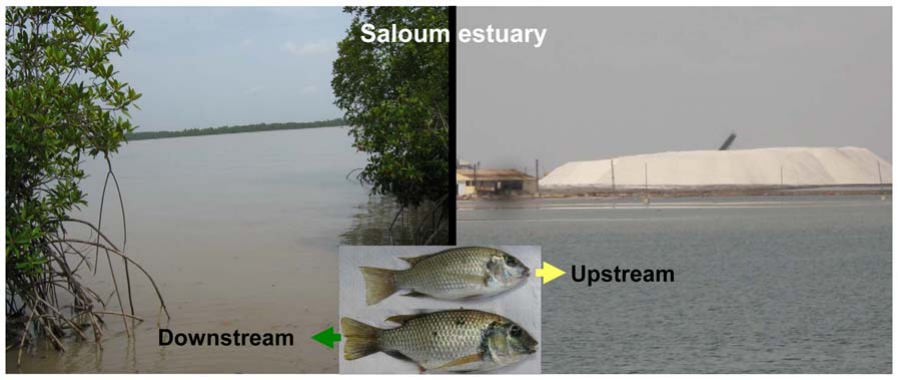
Dissimilarities between downstream and upstream parts of the Saloum estuary (Senegal). The salinity gradient in the estuary is inverted, with lower salinity at the estuary mouth (downstream) than in the upper reaches. The estuary mouth is characterised by a discontinuous mangrove whereas in the upstream part, the mangrove has disappeared because of very high salinities and destructive human activities. Fish inhabiting the upstream part (hypersaline zones) of the estuary have impaired growth performance compared to those living in the downstream zone. Fish analysed in this study were collection in upstream of the Saloum estuary where the salinity is higher.

Hypersaline conditions are increasingly observed in estuarine ecosystems as a consequence of global climate changes. This is especially the case for some Sahelian estuaries in West Africa, where the reduced freshwater influx and water evaporation have resulted in an overall increase of salinity. This region has experienced a succession of drought periods since the 1960's, the impact of which was higher in the estuaries with no or small river connection. In such estuaries, the freshwater inputs which essentially come from groundwater discharges and rainfall, are largely excided by the loss via evaporation [1], [2]. This has resulted in an inversion of the salinity gradient in some estuaries such as the Sine Saloum River (Senegal), with salinities increasing from downstream to upstream where they can exceed 130 psu [3] (Figure 1). The salinity levels in these estuaries also change very significantly between the dry season and the rainy season [3], [4] with amplitudes that can reach 70 psu. Such spatiotemporal variations of salinity constitute serious abiotic obstacles that could deeply impact normal biological function of a species such as growth and reproduction.

Some fish populations have developed adaptive strategies including the regulation of growth and reproduction to cope with these unusually stressful salinity conditions [5], [6], [7], [8]. The reproductive strategy of a fish species is the overall pattern of reproduction common to all individuals and covers a range of life-history traits including the age and size at first sexual maturity, gonadal development, fecundity and gamete size. Individual fish can, however, develop alternative reproductive tactics which are variations with respect to the normal reproduction pattern of the species, to respond to fluctuation in the environment. Both, the overall strategy and tactical variations are adaptive and aim at ensuring the survival of the species in specific environmental conditions [9]. The reproductive tactics or compensations imply that, depending on the environmental conditions, a female can choose between allocating energy to either survival or somatic growth and reproduction. The compensation most commonly involves modifications in fecundity and life span [10]. Roff [11] proposed two models for energy allocation during reproductive compensation: (i) maintenance of body weight and adjustment of gamete production, and (ii) maintenance of a constant number of eggs at the expense of somatic tissues. The first type of compensation occurs when the stress compromises the development of the ovary during vitellogenesis, which can lead to atresia of the eggs and subsequent resorption.

The tilapias are widely distributed in tropical regions where they have colonised a wide range of water bodies as an introduced or native species. Previous studies have demonstrated that the fecundity and egg size in tilapia can be influenced by the food quantity [12], the fishing pressure exerted on the fish [13], [14], but also by environmental conditions [13], [15], [16], [17], [18]. Tilapias are capable of allocating most of their energy reserves to reproduction to the detriment of growth if environmental conditions change [19], [20]. In natural environments a tilapia species can, therefore, show a great variability in its reproductive traits [20], [21]. Among the tilapias, the genus *Sarotherodon* including the species *Sarotherodon melanotheron* and *Sarotherodon galileus* is widely distributed in West-African coastal, estuarine and lagoon ecosystems. The black-chinned tilapia *S. melanotheron* is particularly notable for its ability to tolerate a wide range of environmental salinities [22], [23], [24], [25]. The species is also known to be widely tolerant to temperature variations [3], [4], [26], [27], and dissolved oxygen conditions at the scales of both daily and seasonal fluctuations. Although *S. melanotheron* can tolerate a broad range of salinities, fish inhabiting extremely hypersaline waters of the Sine Saloum estuary exhibited changes in fitness-related traits. It has been demonstrated that black-chinned tilapias living in hypersaline zones of the Saloum (Figure 1) had impaired growth performance and precocious reproduction [4], which have been also reported in another estuarine species, *Ethmalosa fimbriata* [27], [28]. These phenotypic differences have been interpreted as indicative of hypersaline stress.

This study attempted to better understand the mechanisms of salinity adaptation in *S. melanotheron*, in particular the reproductive strategies and/or tactics which may underlie its exceptional euryhalinity. Our study focuses on variation of some reproduction parameters (reproductive period, fecundity and egg size) in natural populations of *S. melanotheron* from three ecosystems with different salinities: Guiers lake (0 psu), Hann bay (37 psu) and upper part of the Saloum estuary (66–127 psu). Reproduction parameters were quantified over two consecutive sampling years. The condition factor of the fish was measured in parallel and taken as a proxy of physiological status. Condition factor is a morphometric index frequently used to evaluate the wellbeing or physiological status of fish, based on the principle that heavier individuals of a particular length which have a higher body weight, are in better “condition”.

## Results

### Correlations between fecundity and body weight

Table 1 shows the relationship between fecundity and the fish body weight. There was a significant and positive correlation between absolute fecundity and fish body weight in the all ecosystems (*P*<0.01). The correlation was significantly stronger (*P*<0.05) in Guiers lake (R^2^ = 0.68; *P*<0.001) compared to Hann bay (R^2^ = 0.33; *P<*0.001) and Saloum estuary (R^2^ = 0.49; *P*<0.001).

**Table 1:** Relationship between fecundity and total body weight.

Relation	Locations	N	a	b	r^2^	*F-*statistic	*df*	*p*
Fecundity-Body weight	Guiers lake	20	1.519	212.174	0.68	37.8	18	***
	Hann bay	53	1.393	673.046	0.33	25.39	51	***
	Saloum	84	4.639	40.069	0.49	77.89	82	***

N = number of observations; *a* and *b* = parameters of the linear regression y = ax+b; r^2^ = coefficient of regression; *df* = number of freedom degree; *p* = probability level; significant levels: * = 0.05; ** = 0.01; *** = 0.001.

### Fish condition factor

**Figure 2:**
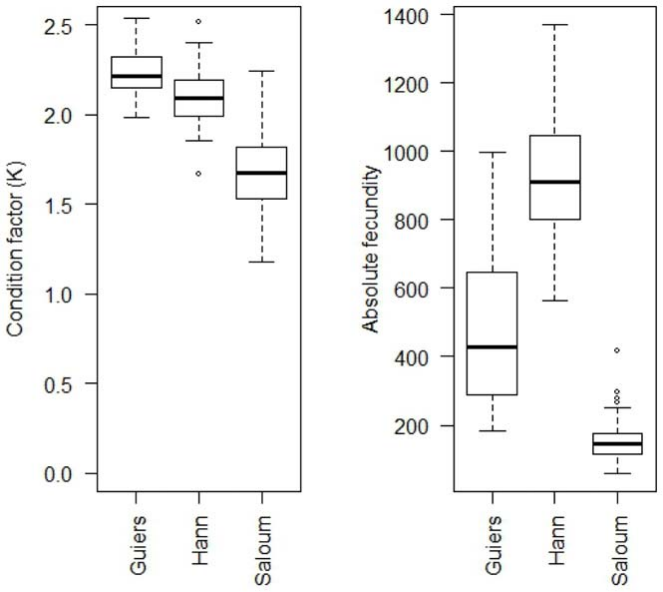
Condition factor and absolute fecundity of the black-chinned tilapia *S. melanotheron* from three wild populations. The condition factor and absolute fecundity were calculated considering samples collected in both years (2003 and 2004) together. Data are illustrated in box plots that contained the median (horizontal line) as well as the 25^th^ and 75^th^ percentiles (bottom and top edges of the boxes).

The average condition factor considering both years together was significantly different between locations or salinities (Figure 2). The best condition factor was observed in fish collected at the freshwater (Guiers lake) and seawater (Hann bay) locations. Fish caught in the Saloum estuary, the most saline location, had the lowest condition, significantly lower than the other sites (ANOVA, *P*<0.05). No significant difference in condition factor was found between Guiers lake and Hann bay (Figure 2). The average condition factor considering the years separately (Table 2) showed similar values in freshwater and seawater and lower values in hypersaline water. There was no significant difference in average condition factor between 2003 and 2004 in each location (Table 2).

**Table 2:** Mean ± (SD) of condition factor and reproductive parameters of the black-chinned tilapia *S. melanotheron* from three wild populations adapted to different environmental salinities.

Ecosystem	Guiers lake		Hann bay		Saloum	
Year	2003	2004	2003	2004	2003	2004
Condition factor	2.34±0.15^a^	2.28±0.19^a^	2.08±0.14^a^	2.13±0.12^a^	1.60±0.14^a^	1.86±0.14^a^
Absolute fecundity	482±202^a^	430±221^a^	868±238^a^	936±276^a^	130±52^a^	168±58^a^
Relative fecundity	2706±215^a^	3241±1235^a^	5843±1520^a^	5308±1824^a^	6899±1884^a^	6111±1524^a^
Oocyte weight (g)	1.58±0.03^a^	1.01±0.31^a^	0.75±0.26^a^	0.80±0.30^a^	0.55±0.23^a^	0.66±0.20^a^
Oocyte diameter (mm)	2.79±0.73^a^	3.14±0.48^a^	2.92±0.45^a^	3.07±0.45^a^	2.67±0.40^a^	2.75±0.32^a^
Egg weight (g)	6.23±4.80^a^	4.89±3.09^a^	7.39±2.87^a^	8.16±3.51^a^	0.84±0.45^a^	1.24±0.39^a^
L_50_±SD (cm)	13.83±0.28^a^	14.66±0.30^a^	11.08±0.37^a^	11.71±0.33^a^	7.84±0.24^a^	7.43±0.44^a^

For each variable, same superscript in the column indicates that there is no significant difference (*P*≥0.05) among years (2003 and 2004) in the same ecosystem.

### Reproductive cycle and size at first maturity

**Figure 3:**
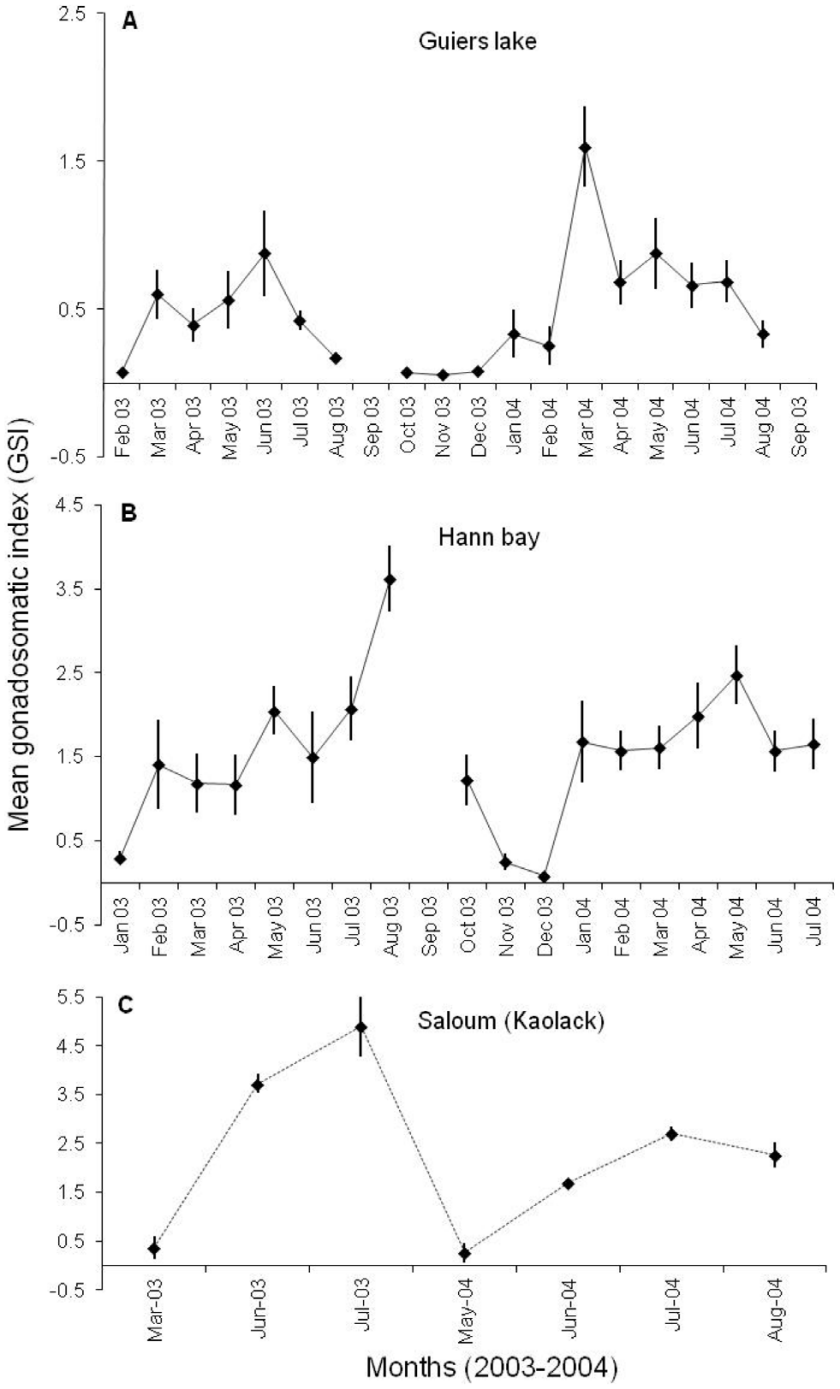
Reproductive cycle of *S. melanotheron* in Guiers lake, Hann bay and Saloum estuary. Data are expressed as the mean GSI ± SD. The space in the curves indicates the lack of samples in September. The reproductive cycle of the species in the Saloum estuary was previously determined by Panfili *et al*. [4]. Therefore, fish were sampled only during the spawning season to confirm the breeding periods determined by these authors.

The reproduction period of *S. melanotheron* in Guiers lake extended from January to August, followed by a period of sexual rest that occurred from October to December (Figure 3A). The peak of sexual activity was recorded in June in 2003 and in March in 2004, suggesting variations in the reproductive cycle over the years. In Hann bay, the reproduction period of the species extended from January to October with a period of sexual rest in November and December (Figure 3B). There were also some differences in the peak of sexual activity, which was observed in August in 2003 and in May in 2004. In each of these ecosystems, the breeding season covered both dry and rainy seasons but the largest part of the reproductive cycle occurred during the dry season. Upstream of the Saloum estuary, the reproductive activity was intense in rainy season (from June to August) when the salinity in the estuary was lower (Figure 3C). Fish sampled in dry season (October to May) was characterized by low gonadosomatic index (*GSI*) and an absence of sexual stages 4, 5 and 6.

Table 2 indicates the size at first sexual maturity of black-chinned tilapia females (*L_50_*) during the reproductive periods in 2003 and 2004. The *L_50_* were higher at Guiers lake location and lower upstream of the Saloum estuary. The *L_50_* at Hann bay location was intermediate between those observed in the two other locations. In each ecosystem, the *L_50_* did not differ significantly between 2003 and 2004.

### Fecundity in black-chinned tilapia populations

**Figure 4:**
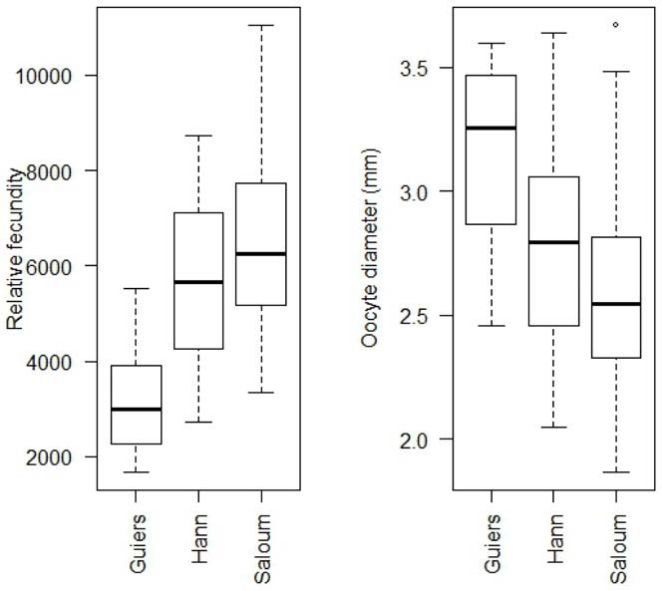
Relative fecundity and oocyte diameter of the black-chinned tilapia *S. melanotheron* from three wild populations. These reproductive parameters were calculated considering samples collected in both years (2003 and 2004) together. Data are illustrated in box plots that contained the median (horizontal line) as well as the 25^th^ and 75^th^ percentiles (bottom and top edges of the boxes).

**Figure 5:**
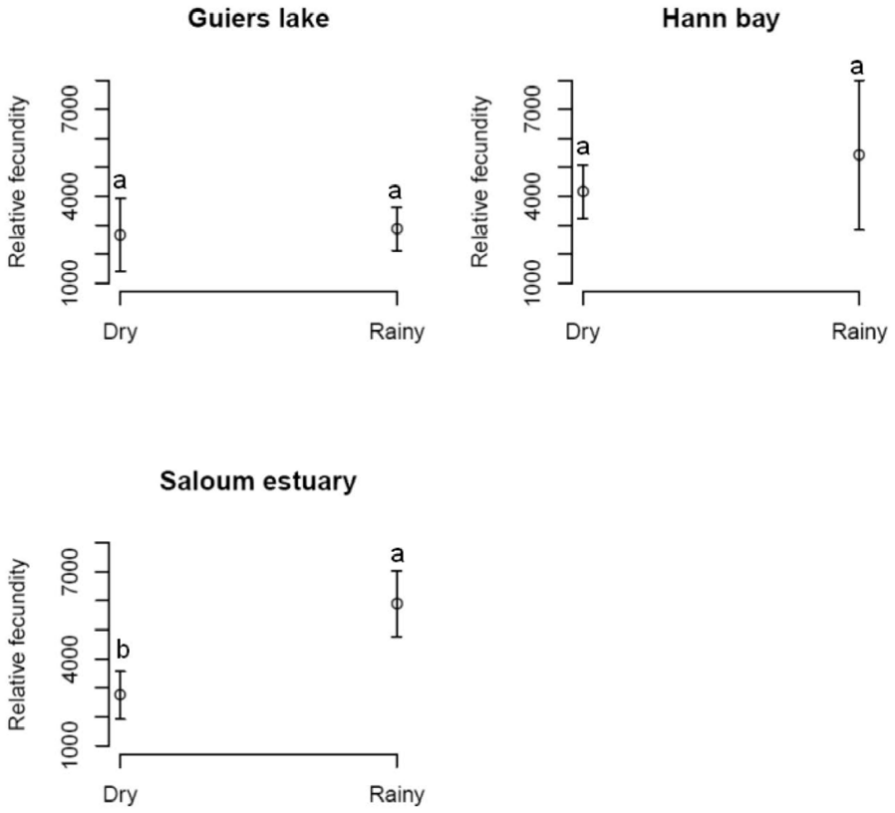
Seasonal variation in relative fecundity in the black-chinned tilapia *S. melanotheron*. Relative fecundity was calculated on females from three wild populations sampled in both the rainy and the dry season. Data are expressed as the mean ± SD.

The average absolute fecundity varied significantly between sites and salinities. Fish sampled in Hann bay had significantly higher absolute fecundity (Kruskal Wallis test; *P*<0.001) than those sampled in the upstream of the Saloum estuary or in Guiers lake (Figure 2). The lowest absolute fecundity was observed in fish sampled in the Saloum estuary. The comparison of absolute fecundity between Guiers lake and the Saloum estuary revealed significantly higher values at Guiers lake. The relative fecundity exhibited a different pattern between locations, being highest at the most saline station of the Saloum estuary (Kruskal Wallis test; *P*<0.001), and lowest at the least saline location, Guiers lake (Figure 4). It did not, however, reveal any significant difference between the Saloum estuary and Hann bay. The average absolute and relative fecundities did not reveal significant differences between 2003 and 2004 in all the ecosystems (Table 2). The relative fecundity was higher in rainy season by comparison to dry season in the Saloum estuary whereas this relationship was not found for the other two locations (Figure 5).

### Oocyte size and total weight of eggs

**Figure 6:**
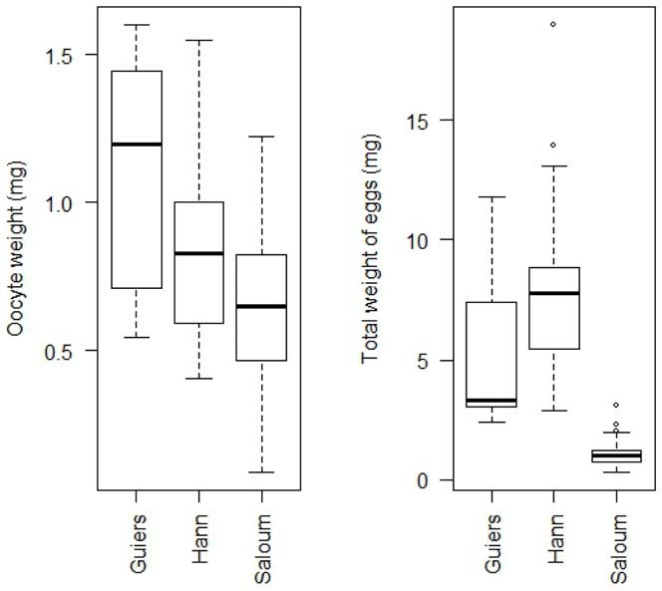
Oocyte weight and total weight of eggs of the black-chinned tilapia *S. melanotheron*. Oocyte weight and total weight of eggs were calculated considering samples collected in both years (2003 and 2004) together. Data are illustrated in box plots that contained the median (horizontal line) as well as the 25^th^ and 75^th^ percentiles (bottom and top edges of the boxes).

Fish sampled in Guiers lake had significantly higher oocyte diameter than those sampled in the Saloum estuary (Tukey test; *P*<0.05) (Figure 4). At Hann bay where the salinity was intermediate, there were intermediate oocyte diameters. The oocyte weight (Figure 6) exhibited a similar pattern between locations, being highest at the Guiers lake, but lowest at the Saloum estuary (Kruskal Wallis test; *P<*0.05). The average oocyte weight at Hann bay location was intermediate to those observed at Guiers lake and the Saloum estuary. By contrast, the total weight of eggs exhibited a different pattern between locations (Figure 6). It is significantly higher at Hann bay than in Guiers lake and Saloum estuary. The average total egg weight was significantly lower at the most saline location. The fish sampled in Guiers lake have total egg weight intermediate to those from the two other locations. The oocyte size (oocyte diameter and weight) and total weight of eggs did not significantly differ between 2003 and 2004 in each ecosystem (Table 2). There was also no significant difference of these parameters between the rainy and the dry season in all locations.

## Discussion

The results demonstrate that the marine *S. melanotheron* population (Hann bay) has significant high absolute fecundity and spawning weight (total weight of eggs) by comparison to those from freshwater or hypersaline water environments. Fish relative fecundity correlates significantly with their total body weight in each environment. The relative fecundity in the Saloum estuary was higher in rainy season compared to the dry season whereas there were no significant seasonal variations in the two other locations. The data also indicate an inverse relationship between relative fecundity and oocyte size in fish living in freshwater and hypersaline water, suggesting different reproductive strategies at these two extreme salinities.

The differences in reproductive traits among populations may reflect environmentally induced phenotypic plasticity and/or genetic variations. In this study we do not have measures of genetic differentiation, but previous studies have revealed high genetic differentiation levels in *S. melanotheron*, even at microgeographical scales [29], [30], suggesting no or very limited gene flow among populations. This high population genetic structure has been assumed to be directly linked to the low larval dispersion ability of the species, due to a mouth-brooding reproductive behaviour. Analyses of strontium/calcium ratios in otoliths also support the sedentary character of *S. melanotheron* [26], which can significantly contribute to population genetic isolation. It can be assumed, therefore, that the *S. melanotheron* populations analysed in this study are genetically different. However, the within-population (upstream Saloum) variability in relative fecundity between the dry and rainy seasons strongly suggests the variations in reproductive traits among *S. melanotheron* populations reflect environmental differences. This conclusion is consistent with those of previous studies [4], [27], [31] that differences in life-history traits of *S. melanotheron* in the Gambia and Senegalese estuaries are related to the environmental salinity rather than genetic differences among populations.

Salinity, temperature and dissolved oxygen are among the abiotic factors which are likely to impact fish fecundity and egg size [13], [15], [16], [17], [18]. The water temperature measured in our study area varied slightly (24.9 to 26.7°C; Table 3) between locations and seasons as previously reported by Simier *et al*. [32] in the Saloum estuary. The amount of dissolved oxygen (DO) is expected to be limiting in the extremely saline environments, because the presence of salt limits the amounts of oxygen that can be dissolved in water. However, previous studies have demonstrated that the level of saturation in DO (81%) of the Saloum water is satisfying [33]. This observation is consistent with the conclusion of other studies that the DO is not a limiting resource in Senegalese estuaries [34]. Therefore, the water salinity is the most constraining environmental factor for *S. melanotheron* in our study area, and seems to be predominant over the other environmental factors.

**Table 3:** Sample characteristics of the black-chinned tilapia *S. melanotheron* and some historical environmental data [48], [49], [50] of the study area.

Variable	Guiers lake	Hann bay	Saloum
Sample size	901	574	862
Min fork length (mm)	48	85	49
Max fork length (mm)	283	292	164
Mean fork length (mm)	191.50±43.93	205.85±30.05	110.47±12.76
Salinity (psu)	0	37	66–127
Water temperature (°C)	24.9	25	26.7
Oxygen (mg l^−1^)	6.70	4.84	-
Transparency (m)	0.79	-	2.5
Conductivity (µs cm^−1^)	174	-	71
Chrorophyll-*a* (µg l^−1^)	23.8	16.83	2.3

### Reproductive cycle of *S. melanotheron*

The black-chinned tilapia *S. melanotheron* exhibited reproductive cycles with an extended intense reproduction period (between 9 and 10 months) in Hann bay and Guiers lake, and a short intense reproduction period (3 months) in the upstream part of the Saloum estuary. These results are congruent with those reported by Panfili *et al*. [4] in the Saloum and the Gambia estuaries. However, in contrast to our results, Legendre and Ecoutin [17] have observed that the reproductive cycle of *S. melanotheron* in Ebrie lagoon (Ivory Coast) is continuous throughout the year with an intense reproductive activity in the dry season. Moreover, Faunce [35] reported that the breeding season of same species in mangrove ecosystems of Florida State (USA) characterised by a semi-tropical climate, extends from April to October. All together, these results indicate that the breeding in *S. melanotheron* is dependent on local environmental conditions. Our results also showed smaller size at first maturity in the upstream part of the Saloum estuary, which can be attributed to the extremely high salinities in this area.

### Among-population variation in fecundity and condition

The absolute fecundity of the black-chinned tilapia was higher in fish adapted to seawater (37 psu) compared to those living in freshwater (0 psu) or hypersaline water (66–127 psu). Likewise, the spawn weight (i.e. total weight of eggs), indicative of reproductive effort, was higher in seawater. Taken together, these results suggest higher reproductive activity in seawater, the optimal salinity for this species [4]. *S. melanotheron* is a marine species of tilapia, which may therefore have evolved mechanisms adapted to have their minimal energetic requirements for maintenance of hydromineral balance in seawater. Such lower energetic cost for osmoregulation would allow supplying more energy to other biological functions, which is evidenced by the best growth performances of *S. melanotheron* in seawater [4], [36]. This interpretation is in accordance with the low expression levels of the gene coding for the Na^+^-K^+^-ATPase 1α pump in fish inhabiting Hann bay compared to those living in Guiers lake and Saloum estuary [37]. Indeed, the Na^+^-K^+^-ATPase is a membrane protein which maintains ion gradients required for cell homeostasis. Therefore, its activity in the gills is related to active ion secretion or absorption in hyper- and hypo-osmotic conditions, respectively. These findings are supported by the poorer condition factors recorded in hypersaline water in this study, although the absence of differences between freshwater and seawater suggest that there may be other factors acting on the fish condition. The specific reasons why fish condition is higher at Guiers lake compared to the Saloum estuary, and did not differ with that of Hann bay are unclear. One possible explanation is that fish in Guiers lake do not need to respond to sudden fluctuations in salinity, which are energetically costly contrary to fish inhabiting the Saloum estuary where the salinity is very unstable. A more likely explanation is that the pollutant loads that characterise the Hann bay negatively impact the fish condition. They may thus interfere with the effects of the salinity and lead to undetectable differences in condition factor between this bay and Guiers lake.

Differences in food resources at sites and/or efficiency at foraging and prey availability can also influence reproductive parameters and fish condition. Food resources could be limited in the environments with highest salinities. Individuals in these areas will invest more in maintenance of hydromineral balance than their counterparts living in seawater with more energy available for other biological functions. While an impact of food resources on fish reproduction and condition cannot be excluded, it is clear that food availability is indirectly related to the salinity which is the main environmental constraint in the Saloum estuary. In fact, the mangrove and its associated fauna are considered as major food resource exploited by fishes in the estuaries [38], [39], [40], [41]. Upstream of the Saloum estuary, the mangrove has disappeared (Figure 1) as a result of very high salinities and destructive human exploitation [42], which may significantly impact the availability of food resources exploited by fish species in these areas. Furthermore, concomitant decreases of growth rate and condition factor with increase of environmental salinity were observed in the Saloum estuary [4], [31], [36], suggesting an indirect impact of hypersaline conditions on food availability.

The highest fecundities recorded in rainy season, when the salinity in the Saloum estuary was lower by comparison to dry season (higher salinity), are an evidence for an impact of environmental salinity on the reproduction in *S. melanotheron*. The reduced salinity in rainy season would lower energy requirement for osmoregulation and, therefore, allow better energy supply to the reproduction. This reproductive tactic of producing large amounts of eggs in the rainy season when the salinity conditions in the estuary are more favourable would ensure a higher survival chance to the larvae and, therefore, lead to a higher larval recruitment.

### Reproductive strategies in *S. melanotheron*

Two reproductive patterns (Figure 4) of *S. melanotheron* can be distinguished from this study: (*i*) low fecundity and a large egg size observed at Guiers lake, and (*ii*) high fecundity with small egg size in Saloum population. Fish collected at Hann bay have relative fecundity and oocyte sizes intermediate to those from the other two sites. It has been demonstrated in fishes that, depending on environmental conditions, females can produce either large number of small eggs or small number of large eggs, due to their flexibility in allocating the energetic reserves between these two modes of reproduction. Many studies have thus reported the existence of trade-off between fecundity and egg size in fishes [43], [44] including tilapia species [13], [15], [16], [17], [18]. In the current study, the high fecundity and small egg size observed in the *S. melanotheron* populations inhabiting the upper Saloum estuary could be an adaptive strategy to extremely high salinities that prevailed in this area. Such a high fecundity would increase the rate of larvae survival and, therefore, enable the species to thrive despite the extremely constraining salinity conditions. Furthermore, small eggs in hypersaline water could decrease the surface exchange between intracellular and ambient mediums and, therefore, reduce the energy expenditure for the maintenance of hydromineral balance (S. Gilles, IRD unpublished data). The interpretation can be, however, different at Guiers lake. Indeed, although freshwater represents an extreme salinity for *S. melanotheron*, the large egg size is in discordance with decrease of hydromineral surface exchange. Low fecundity, large egg and size at first maturity have been suggested to be characteristic of population facing stable environmental conditions. This is because in these habitats, fishes are subjected to high inter-specific competition, which can limit the amount of food available for consumption and lead to high juvenile mortality. In such situation, fishes will maintain egg quality at the expense of the number, and the egg production can therefore vary with individual size. It has been also reported that eggs of large size with a relatively small number is an adaptation to poor food supply for the juveniles [45]. Therefore, in addition to the extreme salinity conditions, the larger egg and lower relative fecundity associated with the larger size at first maturity in Guiers lake where the environmental conditions are relatively stable, could reflect limited food resources.

This study provides new insights into the influence of environmental salinity on life history-traits in *S. melanotheron*, and the findings are somewhat similar to those previously reported by Panfili *et al*. [4]. These latter authors did not, however, analyse fish living permanently in seawater or freshwater, and consequently there is no evidence for difference in life history-traits with fish from hypersaline waters. By including Hann bay and Guiers lake populations to our study, we do not only cover the maximum salinity gradient where *S. melanotheron* is encountered, but we also provide the opportunity of comparing reproductive parameters among populations inhabiting lower and higher salinities and those living at salinities close to iso-osmotic conditions. This has enabled to better highlight the relationship between life history-traits and osmoregulation and assume that these parameters might be in trade-off in *S. melanotheron*. Such a trade-off would occur only if both traits (reproductive parameters and metabolic efforts needed to maintain osmotic homeostasis) are phenotypically plastic in response to salinity variations. Within-population variations in relative fecundity between the dry and rainy seasons in upstream of the Saloum estuary suggest that the latter are environmentally induced. However, further investigations by common garden experiments on variations of life-history traits and osmotic stress indicators in relation to the salinity are needed to establish beyond doubt whether these parameters are plastic.

## Materials and Methods

### Study area

**Figure 7:**
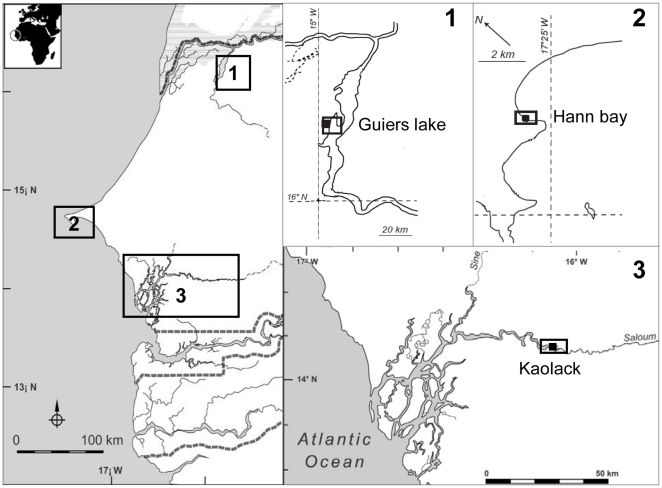
Sampling locations of the black-chinned tilapia *S. melanotheron*. Fish were collected in 2003 and 2004 from three locations in Senegal with different salinities: Guiers lake (0 psu), Hann bay (37 psu) and upstream of the Saloum estuary (Kaolack; 66–127 psu).

With a catchment area of 300 km^2^ at high water, 60 km long and 77 km wide, Guiers lake is located in north Senegal at 18°30W longitude between 15°30 and 16°N latitude (Figure 7). At low water, its catchment area decreases considerably to become 200 km^2^ with a depth from 2 to 3.5 m north and 1 to 1.5 m south. The flat surrounding land, the porous nature of the soils and the low local rainfall greatly reduce the amount of runoff into the lake from its own catchment (not more than 10%). For its water supply, Guiers lake depends mainly on the Senegal River. Before the construction of the dam in Senegal River and the bridge at Richard-Toll in 1947, the Taouey channel brought water from the river to the lake during the flooding and partially emptied it into the river at low water [46].

Hann bay (Figure 7) is about 20 km long and is situated on the Atlantic coast near the Cap Vert peninsula (Dakar, Senegal). It is a site of an important small-scale fishery and is one of the main centers for fishery landings in Senegal. For several years, Hann bay has been affected by eutrophication with a pronounced increase in its nutrient concentrations. Periodic upwellings of cold and nutrient-rich waters occur quite frequently. The combination of this natural nutrient-enrichment phenomenon and the current pollution explains the episodes of blooms of macroscopic algae (Ulvales) and natural phytoplankton [47]. Despite this pollution within the bay, a large quantity of tilapia (mainly *S. m. heudelotii*) is landed every day by the purse seine and especially beach seine fishery.

The Sine Saloum estuary is located between 13°55′ and 14°10′ North and 16°03′ and 16°50′ west and comprises three main branches that are from north to south, the Saloum, the Diomboss and the Bandiala (Figure 7). The estuary drains a catchment of 29,720 km^2^ with a very low slope. The Sine Saloum region is characterized by an extended dry season from November to June, and a short rainy season from July to October. The estuary does not receive freshwater inputs except by precipitation. Therefore, salinity levels change considerably between the rainy season and the dry season [3], [4]. In rainy season, the salinity in the estuary is very unstable and decreases considerable due to the input of freshwater by precipitation whereas it increases during the dry season because of intense evaporation. The combined effect of limited freshwater inputs and intense evaporation has resulted in an overall increase of salinity levels and an inversion of salinity gradient (Figure 1). No specific permits were required to perform experiments in these areas.

### Sampling design

The samples used in this study were collected in accordance with good animal practice as outlined by French Research Institute for Exploitation of the Sea (IFREMER) in a training course on how to handle fish and promote their welfare under experimental conditions. IFREMER do not approve or give a permit for studies of wild populations of fish but only provide a code of conduct to follow to minimize the suffering in experiments involving fish. Study approval by another academic ethic committee (permit number or approval ID) was not necessary as all procedures carried out with the black-chinned tilapia in this study fish are conformed to IFREMER recommendations.

Three natural populations of *S. melanotheron* were sampled in Senegal between January 2003 and August 2004. Two sampling locations, Guiers lake (freshwater) and Hann bay (seawater) do not particularly undergo salinity variations throughout the year (Figure 7). The other sampling location, Kaolack (hypersaline water) located upstream of the Saloum estuary experiences considerable seasonal salinity variations (Figure 7). The reproductive cycle of *S. melanotheron* has never been studied at Guiers lake and Hann bay, therefore fish were monthly sampled in these ecosystems. By contrast, in the Saloum estuary where the reproductive cycle of the species was previously determined by Panfili *et al*. [4], fish were sampled only during the spawning season to confirm the breeding periods determined by these authors. For each location, the salinity and temperature (Table 3) were measured *in situ* with a refractometer (ATAGO) and a thermometer, respectively. Fish sampling was carried out by local fishermen using beach seine net or castnet with small mesh size. All fish were killed by anaesthetization with a lethal dose of 2-phenoxyethanol, and then preserved in 95% ethanol until processing by dissection. In the laboratory, fish were measured (fork length, FL, in mm) and weighed (total mass, W, in g). They were then sexed and the stage of gonad maturity was recorded according to Legendre and Ecoutin [17]. Briefly, the stage 1 corresponds to immature individuals, the stage 2 to beginning of maturation and stage 3 to mature individuals. Stage 4 corresponds to females ready to reproduce, stage 5 to ripe females and stage 6 to post-spawning individuals. The gonads were extracted, weighted and then preserved in 95% ethanol.

### Fish condition factor

The condition factor (K) is a morphometric index frequently used as proxy to evaluate physiological status of fish based on the principle that individuals of a given length, exhibiting higher weight are in better condition. Assuming this relationship, inter population variation of this index has been used to investigate salinity impacts. The condition factor can be influenced by differences in sexual stage. For this reason, we have subtracted the gonad weight from the total body weight to avoid possible bias related to sexual maturity stage. The condition factor was calculated using the remaining weight of samples collected in both years together, but also by considering the two years separately. The condition factor was calculated using the standard formula:
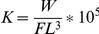
where *W* = body mass without gonads in g and *LF = *fork length in mm

### Gonadosomatic index and size at first maturity

The gonads used for the analysis of fecundity and, the oocyte diameter and weight should be in similar stages of vitellogenic development to avoid any bias in the comparison of egg sizes, which means they should have completed their growth [21]. This implies that the gonadosomatic index (GSI) values should not be positively correlated with the oocyte diameter or weight. Therefore, we classified the gonads into GSI classes of 0.5% for each ecosystem and then determined the GSI threshold above which the oocyte weight and diameter are no longer increasing. This threshold was reached at GSI≥4% in Hann bay and Saloum and at 3% in Guiers lake. The GSI was calculated using the standard formula:
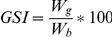
where *W_g_* = gonad weight; *W_b_* = total body weight.

Average size at first maturity (*L_50_*) was defined as the size (the fork Length) at which 50% of individuals in the population reached sexual maturity during the reproduction period. The *L_50_* was determined by modelling the proportion of mature individuals according to their length class for different populations using the R software (http://www.r-project.org). The *L_50_* was estimated using the logistic function expressed by the standard formula:
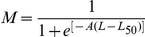
where *M* = percentage of mature females by length class, *L* = central value of the length class and *A* is the constant of the model.

### Fecundity and the egg size

The absolute fecundity is the number of oocytes likely to be released at the next spawning whereas the relative fecundity is the fecundity per kilogram of fish. In *S. melanotheron*, the oocyte distribution in the ovary is multimodal and stage 4 females are more likely to lay oocytes with the highest modal diameter. Therefore, only the stage 4 gonads from females were used to estimate the fecundity. All oocytes in samples were counted manually because the oocytes of *S. melanotheron* are large and easy to manipulate. In upstream of the Saloum estuary, the gonad contained a low number of oocytes, therefore, the whole content was counted. By contrast, at Hann bay and Guiers lake where female gonads contained a large number of oocytes, only a sample of about 50% of the ovary was weighed and counted. The total oocyte count of the gonad was then determined by reporting this weight to the total weight of the gonad.

To determine the oocyte diameter and weight, only those oocytes belonging to the largest size mode in the gonads and whose growth was completed (GSI greater than the threshold) were used. The oocyte diameter was determined using Image J software developed the National Institute of Health (http://rsb.info.nih.gov/nih-image). The software enables processing of digital photos to determine the oocyte number in the sample and the mean oocyte diameter (each being measured along the largest Feret diameter). It also allows determining the minimum and maximum diameter of the oocytes in the sample as well as the oocyte size distribution. As the oocytes are ellipsoidal in *S*. *melanotheron*, the largest and smallest diameters were determined. This was possible because the software fits an ellipse to each egg, giving its major and minor diameters. The major and minor diameters were measured in parallel on an oocyte sample from 15 females using a binocular microscope. Comparison of the results revealed great similarity between the two methods of measurements, which enabled us to validate the method of determining the oocyte diameter by Image J software. The mean weight of an oocyte was determined by weighing 100 oocytes using a mini scales with hundredth gram resolution.

### Statistical analyses

For the fecundity, oocyte diameter, oocyte weight, we performed preliminary analyses of the variance homogeneity and normality of the data using Bartlett and Kolmogorov-Smirnov tests, respectively. One-way ANOVA followed by multiple comparison Tukey test was performed on data of oocyte diameter with normal distribution and uniform variance to test differences between locations. When the data was not normally distributed and did not have uniform variance as for the fecundity and oocyte weight, a Kruskal-Wallis non-parametric analysis of variance (ANOVA) was performed to reveal significant differences in means between locations. Taking all the individual data from the locations, the strength of the correlations between fecundity, oocyte diameter, oocyte weight, egg weight and total body weight were assessed by covariance analysis. These tests were performed with the R software (http://www.r-project.org). For all tests, a probability of less than 5% and a confidence of 95% are considered as fiducial level of significance.
